# Pseudohypoparathyroidism, acrodysostosis, progressive osseous heteroplasia: different names for the same spectrum of diseases?

**DOI:** 10.1007/s12020-020-02533-9

**Published:** 2020-11-11

**Authors:** Francesca Marta Elli, Giovanna Mantovani

**Affiliations:** 1 Endocrinology Unit, Fondazione IRCCS Ca’ Granda Ospedale Maggiore Policlinico, Milan, Italy; 2 Department of Clinical Sciences and Community Health, University of Milan, Milan, Italy

## Abstract

Pseudohypoparathyroidism (PHP), the first known post-receptorial hormone resistance, derives from a partial deficiency of the α subunit of the stimulatory G protein (Gsα), a key component of the PTH/PTHrP signaling pathway. Since its first description, different studies unveiled, beside the molecular basis for PHP, the existence of different subtypes and of diseases in differential diagnosis associated with genetic alterations in other genes of the PTH/PTHrP pathway. The clinical and molecular overlap among PHP subtypes and with different but related disorders make both differential diagnosis and genetic counseling challenging. Recently, a proposal to group all these conditions under the novel term “inactivating PTH/PTHrP signaling disorders (iPPSD)” was promoted and, soon afterwards, the first international consensus statement on the diagnosis and management of these disorders has been published. This review will focus on the major and minor features characterizing PHP/iPPSDs as a group and on the specificities as well as the overlap associated with the most frequent subtypes.

## Introduction

In 1942, Fuller Albright described pseudohypoparathyroidism (PHP), the first known post-receptorial hormone resistance, and not many years passed since he realized the complexity of the disease and recognized the existence of different subtypes. In addition to the best known PHP type 1A (PHP1A) patients, characterized by increased PTH serum levels, hypocalcemia and hyperphosphatemia and a constellation of clinical abnormalities collectively named as Albright Hereditary Osteodystrophy (AHO) (brachydactyly, overweight/obesity, pre and/or postnatal growth retardation, dysmorphic facies with a rounded face, ectopic subcutaneous ossifications, cognitive and/behavioral defects and additional hormone resistances), other patients displayed the presence of physical features of AHO without resistance to the action of PTH and the condition was termed pseudopseudohypoparathyroidism (PPHP) [1–8], while others showed isolated PTH resistance with apparently no other clinical manifestations (PHP type 1B, PHP1B) [9].

The exact prevalence of PHP is unknown. Studies published in 2000 and 2016 estimated the prevalence to be 0.34 in 100,000 in Japan, 1.1 in 100,000 in Denmark and 0.66 in 100.000 in Italy ([10], Orphanet).

The investigation of the pathogenetic mechanism confirmed that the metabolic defect underlying the disease was the lack of responsiveness to the action of PTH in target tissues due to a defective activity of the alpha subunit of the heterotrimeric stimulatory G protein (Gsα) caused by inactivating (epi)genetic defects at the *GNAS* locus [11–20].

The study of the parental transmission of the disease showed that the maternal transmission resulted in the full phenotype with AHO and hormone resistance, while the paternal inheritance was associated with AHO alone or to progressive osseous heteroplasia (POH), a clinical condition defined by the presence of progressive ectopic ossifications [11–22].

The demonstration of the involvement in the disorder of parent-specific tissue-specific imprinting at *GNAS*, leading to decreased Gsα expression in renal proximal tubules and, consequently, to renal resistance to PTH, allowed the identification of the sporadic and the autosomal dominant forms of PHP1B [23–37].

**Fig. 1:**
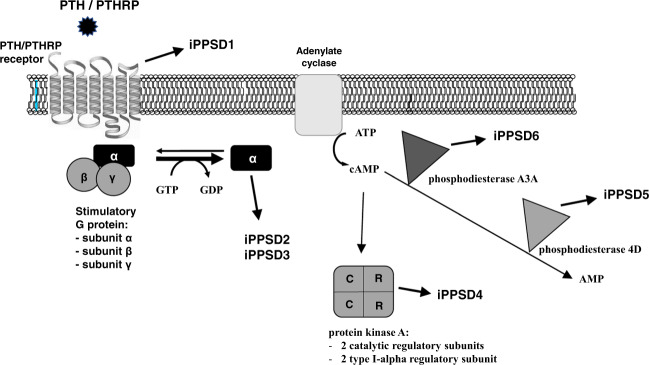
The PTH/PTHrP signaling pathway and associated iPPSD diseases. The figure show proteins involved in the cAMP-dependent intracellular signaling pathway, whose encoding genes, if mutated, are the cause of the reported diseases: the PTH/PTHRP receptor (*PTH1R*), Gsα (*GNAS*), protein kinase regulatory subunit type 1A (*PKAR1A*), the phosphodiesterase 4D (*PDE4D*) and the phosphodiesterase A3A (*PDE3A*)

As research continued on these conditions, it became clear that the above-mentioned picture was complicated by the finding of a clinical and molecular overlap among the different PHP subtypes, as well as by the discovery of genetic defects in genes such as *PRKAR1A* and *PDE4D* associated with acrodysostosis (ACRDYS), which shares many aspects of PHP bone and endocrine phenotype [38–55]. For this reason, in 2016, it was proposed that these conditions should be grouped under the novel term “inactivating PTH/PTHrP signaling disorders (iPPSD)” followed by a numbering specific for each underlying molecular alteration (iPPSD1, loss-of-function mutation in PTH1R; iPPSD2, loss-of-function mutation in *GNAS*; iPPSD3, methylation defects at one or more *GNAS* differentially methylated regions (DMR); iPPSD4, *PRKAR1A* mutation; iPPSD5, *PDE4D* mutation; iPPSD6, *PDE3A* mutation; iPPSDx, no molecular defect identified) [56]. Table 1 summarizes the main clinical and molecular characteristics of these disorders, highlighting the differences between the classical and the novel nomenclature, while Fig. 1 shows the PTH/PTHrP signaling cascade together with diseases associated with mutations of its signaling molecules.

**Table 1:** Main clinical and molecular characteristic of PHP and related diseases

Old classification	New classification	Molecular determinant	Major criteria			Minor criteria					
			PTH resistance	Ectopic ossification	Brachydactyly	TSH resistance	Other hormone resistances	Motor and/or cognitive retardation	Intrauterine and/or postnatal growth retardation	Obesity or overweight	Round face and/or flat nasal bridge and/or maxillar hypoplasia
**BOCD**	**iPPSD1**	*PTH1R* autosomal recessive mutation	No	No	No	No	No	No	Yes	No	Yes
**Eiken**		*PTH1R* autosomal recessive mutation	No	No	No	No	No	Yes	Yes	No	Yes
**PHP1A/PHP1C**	**iPPSD2**	Inactivating maternal *GNAS* autosomal dominant mutation	**Yes**	Yes	**Yes**	**Yes**	Yes	Yes	**Yes**	Yes	Yes
**PPHP/AHO**		Inactivating paternal *GNAS* autosomal dominant mutation	Rare and mild	Yes	**Yes**	Rare and mild	Rare and mild	Yes	**Yes**	No	Yes
**POH**		Inactivating paternal *GNAS* autosomal dominant mutation	No	**Yes**	Rare	No	No	Rare	Yes	No	Rare
**PHP1B**	**iPPSD3**	GNAS sporadic or familial imprinting defects	**Yes**	Rare	Rare	Yes	Rare	No	No	Yes	No
**ACRDYS1**	**iPPSD4**	*PRKAR1A* autosomal dominant mutation	Yes	No	**Yes**	Yes	Rare	Yes	**Yes**	Yes	**Yes**
**ACRDYS2**	**iPPSD5**	*PDE4D* autosomal dominant mutation	Rare	No	*Yes*	Rare	Rare	Yes	**Yes**	Yes	**Yes**
**HTNB**	**iPPSD6**	*PDE3A* autosomal dominant mutation	No	No	Yes	No	No	No	No	No	No

When appropriate, the most frequent clinical signs for each subtypes have been highlighted in bold

*BODC* chondrodysplasia Blomstrand type, *HTNB* hypertension and brachydactyly syndrome

This review will focus on the major and minor features characterizing PHP/iPPSDs as a group and on the specificities associated with *GNAS*, *PRKAR1A* and *PDE4D* alterations. The review will also discuss the variability and clinical overlap among the different subtypes that is often responsible for the delay in obtaining a correct diagnosis.

## PHP/iPPSD clinical features

In 2018 the first international Consensus Statement on the diagnosis and management of these disorders has been published [10]. According to this Statement, the diagnosis of PHP and related disorders are primarily clinical diagnoses and the identification of the molecular cause should be performed subsequently as a confirmation and to allow the characterization of the subtype of the disease. The diagnosis should be therefore based on clinical and biochemical characteristics, which will vary depending on the age of the patient and, in some cases, on the family history. Both the new classification proposal [56] and the Consensus Statement agree that the 3 major criteria are resistance to PTH (rPTH), ectopic ossifications (EO) and brachydactyly (BR), that maybe variably combined in a given patient and associated with other supporting signs and symptoms such as growth impairment (either intrauterine or post-natal), specific face characteristics, obesity, additional hormone resistances, cognitive impairment, mineralization defects such as enamel hypoplasia, delayed tooth eruption or tooth ankylosis, oligodontia or hypodontia, advanced skeletal maturation.

### PTH resistance

PTH resistance is the detection of increased PTH serum levels, hypocalcemia and hyperphosphatemia in the absence of vitamin D deficiency and in the presence of normal magnesium levels and normal renal function. It derives from the unresponsiveness of the renal proximal tubule to PTH. Patients have an increased total, daily, basal urinary excretion of adenosine 3’,5’-monophosphate (cyclic AMP, cAMP), confirming that the metabolic defect underlying the disease is the lack of responsiveness to the action of PTH in target tissues although the presence of abnormally high serum levels of PTH and normal hormone secretion in response to calcium [11, 12].

When present, given the high specificity of this sign, it is sufficient to make a clinical diagnosis of PHP/iPPSD [10, 56].

### Ectopic ossifications

Ectopic ossifications are found on physical examination as palpable hard nodules, whose number location, and extension are highly heterogeneous, showing a predominantly dermomyotomal distribution of lesions. They are often limited to the dermis and subcutaneous tissues, except in POH patients in whom heterotopic bone formation occurs, with an average age-of-onset earlier than 1 year, within the dermis and subcutaneous fat as primary osteoma cutis, then gradually extends to involve deep connective tissues during childhood [22, 57, 58].

As for PTH resistance, this sign is pathognomonic of this group of related diseases and it is thus sufficient to make a clinical diagnosis [10, 56].

### Brachydactyly

In PHP and related disorders, brachydactyly can be classified as type E, which is defined as variable shortening of the metacarpals with, usually, normal length of phalanges, occasionally accompanied by relatively shortened metatarsals. In PHP/iPPSDs the fifth, fourth and third metacarpal and the first and fourth distal phalanges are the most affected bones of the hand but metatarsals are often shortened as well. It is associated with coning of the epiphysis, it is highly variable, often asymmetric, and it usually becomes apparent overtime during infancy/childhood. The identification is based upon the construction of the metacarpophalangeal pattern profile after posteroanterior left-hand radiograph [59]. Brachydactyly may be present in diseases different from PHP/iPPSDs, such as Turner syndrome, the tricho–rhino–phalangeal syndrome and the brachydactyly mental retardation syndrome. It should be therefore accompained by additional major or minor supporting criteria to perform a correct clinical diagnosis [10, 56].

### Minor criteria

Individuals with a more complex phenotype express AHO signs, a series of additional features including resistance to TSH (rTSH), the presence of a dysmorphic facies (flat nasal bridge and/or maxillar hypoplasia and/or round face), obesity or overweight, intrauterine growth retardation (IUGR) and/or post-natal growth retardation, motor and/cognitive retardation or impairment and additional hormone resistances (calcitonin, gonadotropins and growth hormone-releasing hormone), that are considered as minor criteria for the diagnosis [10, 56].

It should be remembered that many are unspecific symptoms that can be also associated with endocrine and syndromic diseases different from iPPSD, and that the number, the age of appearance and the severity of such features vary considerably among patients. Additionally, patients could develop additional features over time or some of them could be really faint and get unnoticed at a first examination.

Resistance to TSH may be detected at neonatal screening, but most patients become clinically resistant to TSH over childhood or adolescence, and, generally, patients display a mild resistance with normal/slightly low thyroid hormone levels in the absence of goiter and antithyroid antibodies [60–62].

Usually, early-onset obesity develops in the first 2 years of life and it is less pronounced in adulthood than in childhood [63–68]. Previous studies observed a low sympathetic nervous system activity, low metabolic rates and low resting energy expenditure, and led to the hypothesis that the weight gain can be associated with a defect in the central nervous system rather than in adipose tissue [69–71].

The PTH/PTHrP pathway plays a pivotal role in the growth plate chondrocytes and the pathogenic *GNAS* monoallelic expression seems to cause an accelerated chondrocyte differentiation and a premature fusion of the growth plate, which in turn determine the lack of pubertal spurt and, consequently, short stature in patients [72–74].

Psychomotor and cognitive abnormalities may be present, defined as a history of developmental delay and learning disability (global retardation of developmental milestones, psychomotor retardation, delayed speech or need of an assistant teacher and extra school help) and the performance IQ seems to be more affected than the verbal IQ [10].

Resistance to additional hormones acting through Gsα-coupled receptors have been also reported in PHP/iPPSD patients, but the clinical relevance of these alterations still needs confirmation. The presence of resistance towards calcitonin was confirmed both in case reports and in a small case series of patients, but nothing is known about the risk of developing medullary thyroid carcinoma [75, 76].

Finally, additional clinical features may be present or evolve throughout time, although they are not required for the diagnosis. They include GH deficiency, hearing impairment, ear infection, spinal stenosis, Chiari malformation type 1 (a condition in which brain tissue extends into the spinal canal), syringomyelia, carpal tunnel syndrome, craniosynostosis, enamel hypoplasia, delayed tooth eruption or tooth ankylosis, oligodontia, or hypodontia, advanced skeletal maturation, cataract, CNS calcifications, sleep apnea, asthma and cryptorchidism [10].

## Specific subtypes

### **GNAS genetic defects: PHP1A/AHO/PPHP/POH or iPPSD2**

The iPPSD2 subtype includes disorders (PHP1A, PPHP, AHO and POH) deriving from inactivating *GNAS* genetic alterations, both point mutations in Gsα-coding exons 1–13 and structural rearrangements (deletions, duplications or inversions affecting part or the whole gene) [10, 56].

Pseudopseudohypoparathyroidism (PPHP) is defined as the presence of the AHO phenotype in the absence of PTH resistance. Patients display a round face, short stature, brachydactyly and ectopic, usually superfical ossifications. Some patients were also originally described as having developmental delay, mild resistance to TSH and obesity [77].

PHP1A is defined by the association of multihormone resistance and an AHO phenotype. PTH resistance and brachydactyly develop over time and become obvious by puberty [78, 79]. Resistance to TSH may be the first manifestation and, being detected in early life or at birth, it may lead to an initial diagnosis of congenital hypothyroidism until brachydactyly and/or PTH resistance become manifest [80]. Even if cognitive impairment has been associated with the diagnosis of PHP1A, normal cognitive development was reported in about 30% of the affected patients [81].

Progressive osseous heteroplasia (POH) describes the condition presenting superficial ossifications progressing into deep connective tissue, with two or fewer AHO features and no PTH resistance [82]. Ossifications cause severe ankyloses of affected joints and focal growth retardation. Associated clinical features supporting the diagnosis are the radiographic evidence for a reticular pattern of ossification, the lateralization of the ossifications in a dermomyotomal pattern, being born small for gestational age (SGA) and leanness.

Typically, PPHP and POH are caused by *GNAS* mutations on the paternal allele, while PHP1A usually displays mutations on the maternal one, leading to the concept that only maternal mutations may cause hormone resistance. Indeed, recent evidence showed that a subset of patients with paternally-derived mutations may develop hormone resistance, and PTH resistance in particular, as well [43].

### GNAS epigenetic defects: PHP1B or iPPSD3

The iPPSD3 subtype includes both forms of PHP1B: the sporadic one, a primary imprinting disorder with a broad *GNAS* methylation defect at all 4 DMRs and the autosomal dominant, a secondary imprinting disorder with a methylation defect limited to the GNAS A/B:TSS-DMR deriving from the deletion of the imprinting control region in the upstream *STX16* gene [23–37]. Initially, PHP1B was defined as an isolated resistance to PTH, but nowadays it is recognized that most patients display additional features, including TSH resistance and patterns of excessive intrauterine growth or weight gain [83, 84]. Moreover, some patients present with one or several features of AHO, brachydactyly being the most frequent [85, 86].

### PRKAR1A and PDE4D genetic defects: ACRDYS type 1 and 2 or iPPSD4 and iPPSD5

iPPSD4 and iPPSD5 are subtypes including acrodysostosis (ACRDYS) caused by *PRKAR1A* and *PDE4D* alterations, respectively. They are defined as the association of severe brachydactyly (usually affecting all phalanxes, metacarpals and metatarsals except thumbs and halluces), facial dysostosis (broad face with widely spaced eyes) and nasal hypoplasia (maxillonasal hypoplasia with flattening of the nasal bridge). Additionally, patients present cone-shaped epiphyses, severe short stature, advanced bone age, hypoplasia of the skull and thickened calvaria, mental retardation, being born small for gestational age (SGA), and resistance to PTH (mainly in patients with *PRKAR1A* mutation) and/or to other hormones signaling through Gsα [45, 49, 52, 87–89]. A small subset of iPPSD5 patients, up to 20%, have an altered response to follicle-stimulating hormone, and cryptorchidism and/or lack of pubertal spurt, possibly secondary to hormone resistance. Additional recurring comorbidities are hearing loss, intracranial hypertension, deformity of knees and shoulders, and atopy/rhinitis/eczema [90].

Again, recent evidence described patients who were clinically diagnosed with PHP1A or PPHP and who then turned out to carry *PRKAR1A* or *PDE4D* mutations [87–89], pointing out the limits of the current, rigid nomenclature.

## Conclusion

The term Pseudohypoparathyroidism-PHP encompasses a group of heterogeneous disorders caused by different genetic and/or epigenetic defects affecting the PTH/PTHrP signaling pathway. The evidence collected over the last decade outlined the limits of the rigid historical classification and a recent proposal suggests to rename an heterogenous but highly overlapping group of diseases encompassing PHP, PPHP, POH and acrodysostosis under the term “inactivating PTH/PTHrP signaling disorders (iPPSDs). Moreover, an international Consensus Statement recently proposed updated recommendations for the diagnosis and management of patients affected with these disorders.

Performing an early and correct diagnosis and a stratification into subtypes is challenging, due to the aforementioned clinical overlap among iPPSD subtypes and the extremely variable presentation and severity of signs and symptoms. For this reason, a multidisciplinary approach is needed and the identification of the underlying genetic or epigenetic defect is fundamental to perform a conclusive diagnosis, allowing an appropriate genetic counseling, treatment, screening for complications and follow-up.
